# Stress CMR T1-mapping technique for assessment of coronary microvascular dysfunction in a rabbit model of type II diabetes mellitus: Validation against histopathologic changes

**DOI:** 10.3389/fcvm.2022.1066332

**Published:** 2023-01-20

**Authors:** Peisong Ma, Juan Liu, Yurou Hu, Lin Chen, Hongqin Liang, Xiaoyue Zhou, Yongning Shang, Jian Wang

**Affiliations:** ^1^Department of Radiology, Southwest Hospital, Army Medical University, Third Military Medical University, Chongqing, China; ^2^Department of Radiology, The First Affiliated Hospital of Chongqing Medical University, Chongqing, China; ^3^Department of Ultrasound, Southwest Hospital, Army Medical University, Third Military Medical University, Chongqing, China; ^4^MR Collaboration, Siemens Healthineers Ltd., Shanghai, China

**Keywords:** adenosine triphosphate, cardiovascular magnetic resonance, coronary microvascular dysfunction, microvascular density, T1-mapping, type 2 diabetes mellitus

## Abstract

**Background:**

Coronary microvascular dysfunction (CMD) is an early character of type 2 diabetes mellitus (T2DM), and is indicative of adverse events. The present study aimed to validate the performance of the stress T1 mapping technique on cardiac magnetic resonance (CMR) for identifying CMD from a histopathologic perspective and to establish the time course of CMD-related parameters in a rabbit model of T2DM.

**Methods:**

New Zealand white rabbits (*n* = 30) were randomly divided into a control (*n* = 8), T2DM 5-week (*n* = 6), T2DM 10-week (*n* = 9), and T2DM 15-week (*n* = 7) groups. The CMR protocol included rest and adenosine triphosphate (ATP) stress T1-mapping imaging using the 5b(20b)3b-modified look-locker inversion-recovery (MOLLI) schema to quantify stress T1 response (stress ΔT1), and first-pass perfusion CMR to quantify myocardial perfusion reserve index (MPRI). After the CMR imaging, myocardial tissue was subjected to hematoxylin-eosin staining to evaluate pathological changes, Masson trichrome staining to measure collagen volume fraction (CVF), and CD31 staining to measure microvascular density (MVD). The associations between CMR parameters and pathological findings were determined using Pearson correlation analysis.

**Results:**

The stress ΔT1 values were 6.21 ± 0.59%, 4.88 ± 0.49%, 3.80 ± 0.40%, and 3.06 ± 0.54% in the control, T2DM 5-week, 10-week, and 15-week groups, respectively (*p* < 0.001) and were progressively weakened with longer duration of T2DM. Furthermore, a significant correlation was demonstrated between the stress ΔT1 vs. CVF and MVD (*r* = −0.562 and 0.886, respectively; *p* < 0.001).

**Conclusion:**

The stress T1 response correlated well with the histopathologic measures in T2DM rabbits, indicating that it may serve as a sensitive CMD-related indicator in early T2DM.

## Introduction

Type 2 diabetes mellitus (T2DM) directly affects the myocardium structure and function and increases the risk of adverse cardiovascular events, even in the absence of hypertension or coronary artery disease (CAD) (1–4). Coronary microvascular dysfunction (CMD), featuring pathologically diminished microvascular dilation capacity in response to increased demand, is prevalent in T2DM and predisposes to increased risk of more severe cardiovascular events (5–7). A previous study reported comparable cardiac mortality for individuals with obstructive CAD and those with non-obstructive CAD but impaired coronary vascular function among T2DM patients (8). Therefore, accurate diagnosis of CMD is crucial in guiding clinical management. Currently, indices such as reduced coronary flow reserve (CFR) and microcirculatory resistance index (IMR) are performed to identify CMD (9–11); however, both techniques often require invasive angiography.

The non-contrast stress T1 mapping on CMR has recently been demonstrated to accurately depict myocardial ischemia and microvascular dysfunction (12–15). Myocardial T1 values, as measured by T1-mapping, are elevated in conditions linked to increased myocardial water content (16, 17), which may be of intracellular or extracellular origin, including the interstitial and intravascular compartments (18). Stress T1 response (stress ΔT1) is defined as the percentage increase in native T1 values from rest to vasodilation stress, capable of reflecting subtle alternations in myocardial blood volume (MBV) (9, 19). Previous studies have shown that the stress ΔT1 was impaired in patients with myocardial ischemia in the absence of obstructive CAD (15, 20). Although a correlation between T1 response and microcirculation function has been proven, it remains to be explored that whether CMR T1 mapping technique can reflect microvascular structural alterations related to T2DM.

In this study, we sought to evaluate the histopathologic alternations relevant to CMD in a T2DM rabbit model more comprehensively, and validate the efficiency of the stress T1 response to adenosine triphosphate (ATP) acquired using T1 mapping at three Tesla CMR for identifying CMD against histopathologic features. Additionally, changing trends of CMD-related parameters were observed by comparing the CMR indices and histopathologic findings among the T2DM rabbits.

## Materials and methods

### Experimental model

All experimental protocols were approved by the Laboratory Animal Welfare and Ethics Committee of the Army Military Medical University in Chongqing, China. New Zealand white rabbits (*n* = 38) with an average weight of 2.5 kg were randomly assigned to four groups: Control (*n* = 8), T2DM 5 weeks (*n* = 10), T2DM 10 weeks (*n* = 10), and T2DM 15 weeks (*n* = 10). Rabbits in the T2DM group were fed a high-fat diet (HFD) for 8 weeks and given a single injection of alloxan (100 mg/kg; APE x BIO, America) into the ear vein after fasting for 12 h, followed by administration of 5% glucose over 12 h to avoid insulin shock and a continuous supply of HFD and liquids. The same dose of saline was injected into the rabbits of the control group. Animals with fasting blood glucose values higher than 11.1 mmol/L for more than three successive weeks were considered successful.

### Cardiac magnetic resonance protocol

Images were acquired on a 3.0-Tesla MR scanner (MAGNETOM Trio, Siemens Healthineers, Erlangen, Germany) equipped with a commercially 8-channel Rabbit Cardiac coil (Suzhou Medcoil Healthcare, Jiangsu Province, China) and ECG gating. The rabbits were anesthetized with 30–40 mg/kg of pentobarbital to reduce heart rate and basal metabolic rate. Animals were fasted for 12 h prior to scanning and an intravenous channel was opened in the ear vein.

**FIGURE 1:**
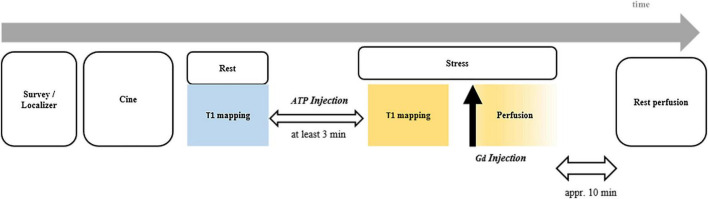
Timeline for CMR scan protocol. CMR, cardiac magnetic resonance.

Cardiac magnetic resonance protocol is shown in Figure 1. Left ventricle (LV) long-axis images and a stack of successive short-axis slices covering the entire LV were obtained with balanced steady-state free precession (bSSFP) sequence. For myocardial T1 mapping, an optimized bSSFP-based modified Look-Locker inversion recovery (MOLLI) with a 5b(20b)3b schema was adopted to minimize heart rate dependency, as previously described (21, 22). The detailed scanning parameters were previously described (22). Briefly, T1-maps were obtained at rest and during peak vasodilator stress (ATP; intravenous at 140–210 μg/kg/min for at least 3 min) in the mid-ventricular short-axis slice (23, 24). Stress first-pass perfusion imaging was performed on matching short-axis slice to the T1-maps during peak stress with an intravenous bolus of gadolinium (0.2 mmol/kg; Dotarem, Guerbet, BP7400, F95943, Roissy CdG Cedex, France) as previously described (22). Resting perfusion images were acquired at least 10 min after stopping ATP.

In this study, the minimum threshold of stress was defined as the following criterion: Heart rate (HR) increases of at least 10 beats per minute. HRs were observed at 1-min intervals beginning with the third minute using a 3-lead electrocardiogram (ECG). When the above criteria were met, stress first-pass perfusion imaging was immediately performed. If the alteration in heart rate was insufficient, a subsequent 50% increased infusion rate would be given to achieve an appropriate hemodynamic response.

### Cardiac magnetic resonance image analysis

**FIGURE 2:**
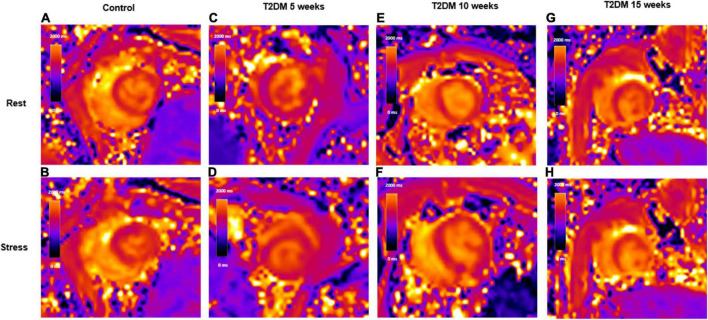
Schematic representation of rest and stress T1 maps. A control rabbit with a resting T1 map, 1,355 ms **(A)** and corresponding stress T1 map, 1,445 ms **(B)**; A 5-week T2DM rabbit with a resting T1 map, 1,321 ms **(C)** and corresponding stress T1 map, 1,379 ms **(D)**; A 10-week T2DM rabbit with a resting T1 map, 1,378 ms **(E)** and corresponding stress T1 map, 1,426 ms **(F)**; A 15-week T2DM rabbit with a resting T1 map, 1,429 ms **(G)** and corresponding stress T1 map, 1,469 ms **(H)**. Values in the graphs indicate the median T1 values of myocardial interventricular septum. T2DM, type 2 diabetes mellitus.

All scans were anonymized and analyzed offline by blinded observers. Short-axis cine images were used to acquire conventional cardiac function parameters by drawing epi-and endocardial contours using the commercial post-processing software cvi42 (Circle Cardiovascular Imaging Inc., Calgary, Alberta, Canada) (25). For rest and stress T1-map quantification, regions of interest (ROI) were manually localized within the myocardium of the interventricular septum as previously described (22). ROIs were carefully matched between rest and stress T1-map images. Stress T1 reactivity was presented in% as: (T1 stress–T1 rest)÷T1 rest × 100 (Figure 2). The intra- and interobserver variability of the rest- and stress T1 measurement were analyzed.

For perfusion analysis, all ATP-induced stress perfusion images were systematically screened by two experienced radiologists for the presence or absence of perfusion defects to rule out potential CAD, as described in previous studies (26). Perfusion defects were identified positive when a perfusion delay persisted for at least five consecutive phases in at least one segment or more on stress perfusion images. Furtherly, signal intensity curves were reconstructed by manually sketching endocardial and epicardial contours to yield MPRI. MPRI was defined as the ratio of stress to the rest upslope, normalized to the blood-pooled time–signal intensity curve (27).

### Biomedical measurements

Blood was obtained by lateral ear vein prick of conscious rabbits at the end of experiments. Fasting blood glucose and lipid profiles, including triglycerides, total cholesterol (TC), high-density lipoprotein (HDL), and low-density lipoprotein (LDL) were measured with a fully automated biochemical analyzer (Shenzhen Meizu Biomedical Electronics Co., Ltd, China). Insulin was measured by ELISA kits (Rexin Biologicals, China). Myocardium tissues of the interventricular septum were weighted and homogenized in an appropriate buffer using an electrical homogenizer. The tissue homogenates (10% mass concentration) were centrifuged at 10,000 rpm for 10 min at 4°C and supernatants were separated. Finally, the oxidative stress indicators were measured accordingly, including the level of nitric oxide (NO) and malondialdehyde (MDA), as well as the expression of glutathione peroxide (GSH-PX) and superoxide dismutase (SOD), by using commercially available kits (Nanjing Jiancheng Corp., Nanjing, China).

### Histopathologic analysis

After MR scanning, hearts were harvested for histological analysis as previously described (22). Briefly, hearts were formalin-fixed and subsequently sectioned across their myocardium of the interventricular septum, matching the ROIs extracted from T1 maps. These sections were subjected to hematoxylin-eosin (H&E), Masson’s trichrome, and CD31 staining, respectively. Microvascular density (MVD) and collagen volume fraction (CVF) were quantified as previously described (22, 28), using Image-Pro Plus 6.0 analysis software (Media Cybernetics, Silver Spring, MD). Briefly, the stained sections were screened at 40 magnification to identify the areas of highest CD31-positive vessel density and these areas were then counted at 200 magnification in six random fields for each animal model. The number of microvessels in each field was determined and expressed as the MVD. The CVF was calculated as the mean collagen content in six randomly selected fields (magnification ×200) of the Masson’s trichrome-stained sections (CVF = total collagen area÷the total image area). Histopathologic findings were analyzed by two pathologists with more than 5 years working experience.

In addition, a piece of left ventricular apical tissue, approximately 1 mm × 1 mm × 1 mm in volume, was placed in 3% glutaraldehyde and fixed in 1% osmium acid, dehydrated in acetone, replaced with propylene oxide, embedded in epoxy resin, and sectioned to observe the ultrastructure of the myocardium under transmission electron microscopy.

### Statistical analysis

Analysis was conducted using GraphPad Prism (version 6.01, GraphPad Software, Inc., La Jolla, California, USA) and MedCalc (version 13.3.3.0, MedCalc Software, Ostend, Belgium). Data are presented as mean ± SD for all parametric data and were checked for normality using the Shapiro–Wilks test. Differences between groups were performed using one-way analysis of variance (ANOVA) tests. When appropriate multiple comparisons were adjusted for using the Tukey method. Correlations between variables were analyzed using the Pearson (bivariate) correlation. Intraclass correlation coefficients (ICC) were calculated to assess intra- and interobserver variation. For all statistics a *p* < 0.05 was considered statistically significant.

## Results

**FIGURE 3:**
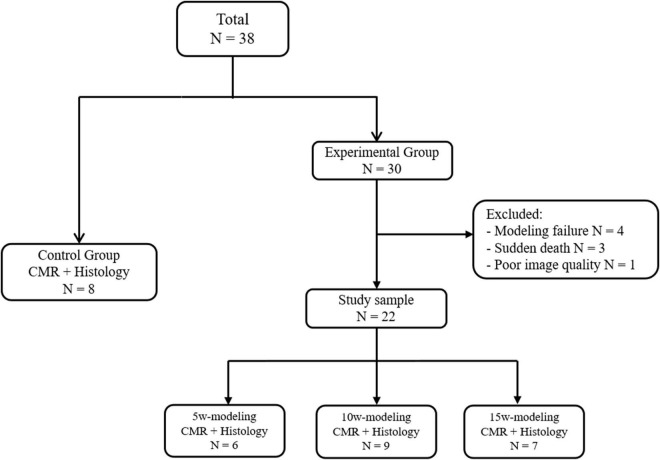
Flowchart of the study design. CMR, cardiac magnetic resonance.

A flowchart of the study design is shown in Figure 3. In the T2DM group, three rabbits died within 24 h of receiving the alloxan injection, and four rabbits gradually returned to normal blood glucose levels, and one rabbit was excluded due to poor image quality during CMR. Thus, a total of 30 rabbits have been included in the analysis: Eight control rabbits, six 5-week T2DM rabbits, nine 10-week T2DM rabbits, and seven 15-week T2DM rabbits.

### Biochemical parameters

As shown in Table 1, fasting blood glucose, insulin, and insulin resistance index levels in T2DM rabbits were significantly higher than those in the control group (*p* < 0.001). Additionally, triglycerides and TC levels in the T2DM group were significantly raised compared to the non-T2DM group (*p* < 0.001), but there were no significant differences among the groups for serum HDL or LDL levels (*p* = 0.281 and 0.359, respectively).

**TABLE 1:** Biochemical parameters in controls vs. T2DM groups.

	Control group	T2DM 5 weeks	T2DM 10 weeks	T2DM 15 weeks	*P*-value
Glucose, mmol/L	5.71 ± 0.96	20.82 ± 8.47*	17.93 ± 7.73*	28.74 ± 8.01*^‡^	<0.001
Insulin, mU/ml	12.09 ± 0.49	23.23 ± 6.28*	19.15 ± 1.51*	18.02 ± 1.16*	<0.001
Insulin resistance index	3.17 ± 0.60	23.18 ± 10.12*	14.29 ± 5.42*	25.91 ± 2.00*^‡^	<0.001
**Blood lipid profile**					
Triglycerides, mmol/L	1.04 ± 0.15	7.65 ± 1.98*	6.65 ± 1.13*	5.19 ± 1.79*^†^	<0.001
Total cholesterol, mmol/L	1.15 ± 0.22	27.69 ± 8.38*	22.99 ± 9.84*	26.06 ± 11.41*	<0.001
HDL, mmol/L	0.52 ± 0.10	0.49 ± 0.09	0.49 ± 0.12	0.47 ± 0.09	0.836
LDL, mmol/L	0.36 ± 0.11	0.44 ± 0.07	0.35 ± 0.08	0.39 ± 0.10	0.888
**Oxidative stress**					
NO, μmol/L	0.84 ± 0.90	0.22 ± 0.08*	0.23 ± 0.10*	0.23 ± 0.10*	0.005
SOD, U/mg protein	6.43 ± 2.40	3.00 ± 1.12*	2.99 ± 0.88*	2.72 ± 1.50*	<0.001
GSH-PX, U/mg protein	105.80 ± 38.72	57.28 ± 27.58*	77.78 ± 13.05*	68.28 ± 23.80*	0.023
MDA, nmol/mg protein	0.46 ± 0.16	1.96 ± 0.91*	1.86 ± 1.08*	1.63 ± 0.40*	0.015

GSH-PX, glutathione peroxide; HDL, high density lipoprotein; LDL, low density lipoprotein; MDA, malondialdehyde; NO, nitric oxide; SOD, superoxide dismutase; T2DM, type 2 diabetes mellitus. Values are mean ± SD.

*P < 0.01 compared with control subjects.

^†^*P* < 0.01 compared with T2DM 5 weeks subjects.

^‡^*P* < 0.01 compared with T2DM 10 weeks subjects.

The changes of myocardial oxidative stress parameters in rabbits are shown in Table 1. Compared with controls, significant decreases in NO, SOD, and GSH-PX concentrations were observed in T2DM rabbits (*p* = 0.005, 0.001, and 0.023, respectively), while MDA increased significantly (*p* = 0.015).

### Cardiac function data

The measured cardiac function parameters are presented in Table 2. ESV was significantly increased in T2DM 10 week and T2DM 15-week groups compared to controls. But there were no significant differences in EF, EDV, SV, or CO between groups.

**TABLE 2:** Cardiovascular magnetic resonance results in controls vs. T2DM groups.

	Control group	T2DM 5 weeks	T2DM 10 weeks	T2DM 15 weeks	*P*-value
EF (%)	53.87 ± 4.61	52.48 ± 4.08	51.7 ± 2.12	50.79 ± 2.04	0.355
EDV (ml)	3.88 ± 0.66	4.42 ± 0.93	4.98 ± 1.00	4.84 ± 0.79	0.066
ESV (ml)	1.77 ± 0.245	2.11 ± 0.43	2.40 ± 0.47*	2.39 ± 0.47*	0.017
SV (ml)	2.11 ± 0.47	2.34 ± 0.55	2.58 ± 0.56	2.45 ± 0.32	0.260
CO (L/min)	0.28 ± 0.66	0.32 ± 0.08	0.34 ± 0.07	0.33 ± 0.04	0.277
Rest T1 (ms)	1,340 ± 15.5	1,340 ± 19.85	1,351 ± 21.62	1,418 ± 29.87*^†‡^	<0.001
Stress T1 (ms)	1,423 ± 18.51	1,406 ± 25.39	1,402 ± 22.40	1,461 ± 27.02*^†‡^	<0.001
MPRI	1.91 ± 0.26	2.03 ± 0.19	1.77 ± 0.16	1.63 ± 0.17^†^	0.007
Stress ΔT1 (%)	6.21 ± 0.59	4.88 ± 0.49*	3.88 ± 0.40*^†^	3.06 ± 0.54*^†‡^	<0.001

CO, cardiac output; EDV, end-diastolic volume; EF, ejection fraction; ESV, end-systolic volume; MPRI, myocardial perfusion reserve index; SV, stroke volume; T2DM, type 2 diabetes mellitus. Values are mean ± SD.

*P < 0.01 compared with control subjects.

^†^*P* < 0.01 compared with T2DM 5 weeks subjects.

^‡^*P* < 0.01 compared with T2DM 10 weeks subjects.

### Histologic results

**FIGURE 4:**
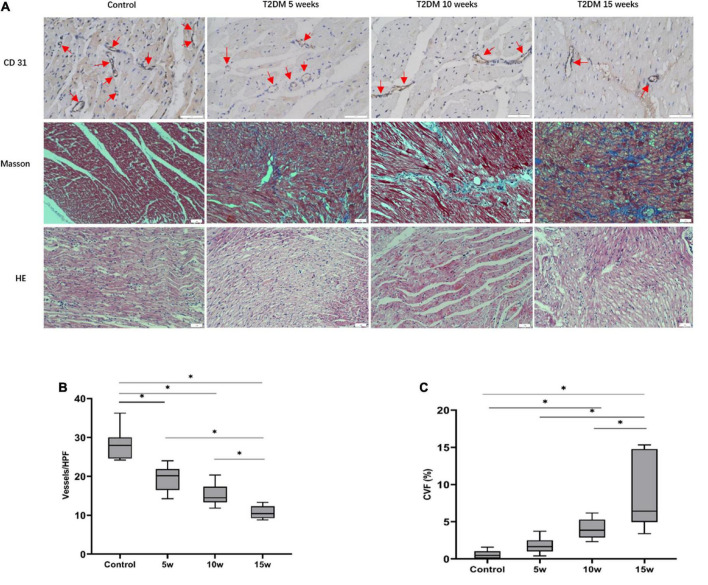
Histopathologic analysis of myocardial alterations in the interventricular septum. **(A)** CD31 staining (scale bars = 50 μm): With increasing durations of T2DM, the number of micro-vessels gradually declined (red arrows). Masson staining (blue, fibrosis, red, myocardial fibers; scale bars = 50 μm); with increasing durations of T2DM, increase in interstitial fibrosis are seen. Histological changes of heart tissue (scale bar = 50 μm). A quantitative analysis of the **(B)** microvascular density (MVD) and the **(C)** collagen volume fraction (CVF) of six random fields at ×200 magnification. The data are expressed as the means ± SD. HPF, high power field; T2DM, type 2 diabetes mellitus.

A representative image to illustrate the histopathological alterations in each of the four groups (control, T2DM 5 weeks, 10 weeks, and 15 weeks) is displayed in Figure 4. Myocardial fibers in the control group were regularly arrayed and cardiomyocytes exhibited morphological normality. In contrast, the rabbits in the T2DM groups demonstrated disarranged and elongated myocardial fibers, as well as cellular oedema. In addition, electron microscopy shows slight swelling of the mitochondria in T2DM rabbits (Supplementary Figure 1).

The average MVD values among the four groups were 28.18 ± 3.97 for control, 19.50 ± 3.37 for T2DM 5 weeks, 15.39 ± 2.72 for T2DM 10 weeks, and 10.75 ± 1.67 for T2DM 15 weeks. In the T2DM groups, a longer duration of diabetes resulted in a lower MVD (Control vs. T2DM 5 weeks, *p* < 0.001; T2DM 10 weeks vs. T2DM 15 weeks, *p* = 0.028, Figure 4). Although there was no statistical difference in MVD between T2DM 5 weeks and T2DM 10 weeks groups (*p* = 0.075), it was trending downward in the latter. Further, the severity of diffuse interstitial fibrosis as well as perivascular fibrosis increased as the increased duration of diabetes in the T2DM groups, as confirmed by Masson trichrome staining. The CVF values in the control, T2DM 5 weeks, 10 weeks, and 15 weeks were 0.58 ± 0.56%, 1.79 ± 1.14%, 4.02 ± 1.32%, and 8.28 ± 4.83%, respectively (*p* < 0.001). The mean CVF in group 10 weeks (*p* = 0.041) and 15 weeks (*p* < 0.001) were significantly higher than in control rabbits (Figure 4).

### T1-mapping Analysis and its relation to pathology

**FIGURE 5:**
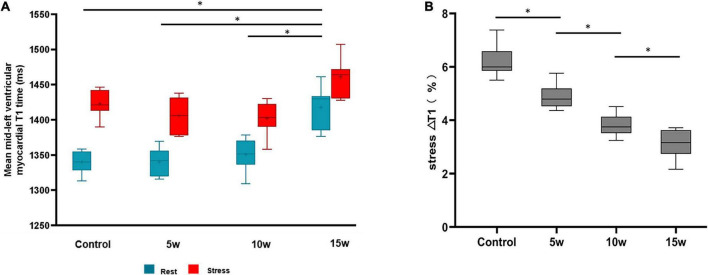
Differences at rest vs. stress of LV myocardial T1 values between healthy controls and rabbits with T2DM **(A)**. The myocardial T1 responses to ATP stress (stress ΔT1) **(B)**. The data are expressed as the means ± SD. LV, left ventricular; T2DM, type 2 diabetes mellitus.

Rest- and stress- T1 values are shown in Table 2. The rest- and stress- T1 values in group T2DM 15 weeks were significantly higher compared to controls (*p* < 0.001), group 5 weeks (*p* < 0.001), and group 10 weeks (*p* < 0.001), as shown in Figure 5. Stress ΔT1 values were 6.21 ± 0.59%, 4.88 ± 0.49%, 3.80 ± 0.40%, and 3.06 ± 0.54% in control, T2DM 5-week, 10-week, and 15-week groups, respectively (*p* < 0.001) and were significantly attenuated in T2DM rabbits with a longer duration of diabetes (Control vs. T2DM 5 weeks, *P* < 0.001; T2DM 5 weeks vs. T2DM 10 weeks, *P* = 0.002; and T2DM 10 weeks vs. T2DM 15 weeks, *P* = 0.036, Figure 5). Besides, the MPRI value in group T2DM 15 weeks was lower compared to group 5 weeks (*p* = 0.006).

The Bland–Altman plotting of rest and stress T1 mapping were shown in Supplementary Figure 2. The ICC coefficients indicated a high degree of consistency for inter- and intra-observer assessments (ICC = 0.800 and 0.839, respectively; *p* < 0.001).

**FIGURE 6:**
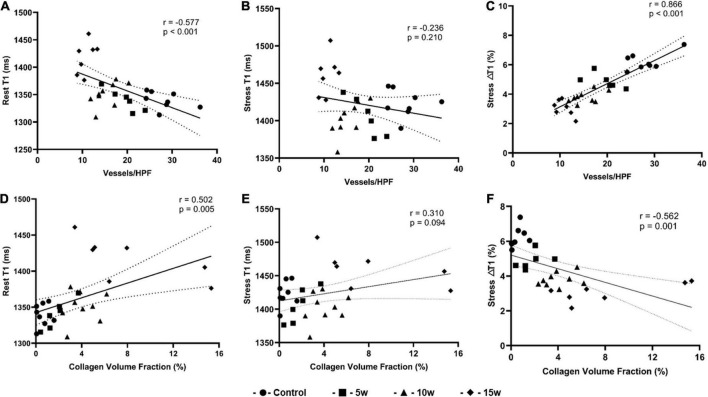
Graphs show correlation between histopathologic findings and imaging parameters derived from T1 mapping for all four groups (*n* = 30). **(A)** Rest T1 values and MVD correlation; **(B)** Stress T1 values and MVD correlation; **(C)** Stress T1 response and MVD correlation; **(D)** Rest T1 values and CVF correlation; **(E)** Stress T1 values and CVF correlation; **(F)** Stress T1 response and CVF correlation. CVF: collagen volume fraction; MVD: microvascular density.

As illustrated in Figure 6, the rest T1 value exhibited a moderate correlation with MVD (*r* = –0.577, *p* < 0.001) and CVF (*r* = 0.502, *p* = 0.007), whereas no significant correlations were observed between stress T1 value against MVD (*r* = –0.226, *p* = 0.210) and CVF (*r* = 0.310, *p* = 0.094). In addition, stress ΔT1 showed a high correlation with MVD (*r* = 0.886, *p* < 0.001) and a moderate correlation with CVF (*r* = −0.562, *p* = 0.001).

## Discussion

Recently, the use of stress T1 mapping on CMR to determine CMD among T2DM individuals has attracted increasing attention (29). Although previous studies have confirmed the stress T1 response can serve as an imaging marker to observe abnormal microvascular function, histopathology studies are still lacking and the potential mechanism underlying this technique remains to be elucidated. Our study performed serial assessments against histologic analysis at various time points in an animal model of T2DM, and the variations in CMR were compared with histological results. The main findings of this study were:

(1) Stress ΔT1 obtained from T1 mapping was significantly related to histopathologic measures, including MVD and CVF;
(2) Stress T1 responses were gradually weakened among rabbits with a longer duration of T2DM.

Coronary microvascular dysfunction in T2DM involves a wide range of pathophysiological changes associated with cardiomyocyte hypertrophy (30), myocardial microvascular rarefaction (31), and perivascular or interstitial fibrosis (32). Persistent exposure to hyperglycemia, hyperlipidemia, hyperinsulinemia, and insulin resistance can induce oxidative stress of microvascular endothelial cells, contributing to impaired microvascular dilatation capacity due to low NO bioavailability (33–35). This study constructed a well-developed rabbit model of T2DM, featuring elevated serum glucose, triglyceride, cholesterol, and insulin concentrations and a higher insulin resistance level compared with control rabbits. Altered indicators of oxidative stress in myocardial tissue, such as decreased NO, SOD, and GSH-PX along with increased MDA, implied potential impairment of microvascular vasodilation capacity. In addition, structural changes in myocardial microcirculation were also found in T2DM rabbits in terms of diminishing microvascular density and increased myocardial fibrosis.

Coronary microvascular dysfunction is an early feature of diabetes (36) and has emerged as a potential contributor to adverse cardiovascular event risk (37). Thus, early identification of CMD might be beneficial for clinical risk assessment and personalized therapy. It was found that the stress T1 responses were gradually attenuated with increased duration of insulin resistance, indicating that stress T1 mapping may be an acceptable non-invasive surrogate to define CMD with high accuracy. The absence of stress response seen in corresponding areas may depend on the blunted microvascular vasodilatory reserve. In addition, this study observed a decrease in MPRI in the 15-week group compared to the 5-week group, while there were no significant differences amongst the remaining groups. This could be explained by the idea that myocardial perfusion regulation is intricate and driven by a multitude of factors, such as blood pressure, metabolic demand, and diastolic time (26, 38). Thus, all factors should be taken into comprehensive consideration when assessing MPRI, which also implies that the sensitivity of stress perfusion based semi-quantitative method was probably lower than that of stress T1 mapping in reflecting subtle changes for microvascular blood flow during the early stage of diabetes.

Well-defined correlations between stress T1 response and pathologic measurements of MVD and CVF were verified, revealing that the abnormalities in microvascular vasodilatory function are associated with myocardial structural changes in CMD rabbits. Although the rest and stress T1 values in the current study did not differ significantly from controls until week 15, the changes in T1 values began to decline immediately at week 5. Microvascular dysfunction has been shown to be a significant contributor to overt cardiac dysfunction, such as increased LV mass, concentric geometry, and early preclinical systolic and diastolic dysfunction; thus, the stress T1 response may serve as a clinically viable indicator for improving coronary microvascular in the early stages of diabetes/prediabetes. In addition, two different types of microvascular dysfunction have been defined: Functional CMD primarily driven by elevated resting blood flow in the presence of normal IMR and structural CMD caused by increased microvascular resistance (39), both of which reflect impaired coronary flow reserve in diabetic patients (40). Although the above studies provide a new insight into CMD, structural CMD is indirectly determined based on hemodynamic indices from percutaneous coronary angiography (PCI) or echocardiography. In the present study, microcirculatory structural changes in T2DM rabbits were shown through histopathological analysis and found to be closely related to the stress T1 response. However, due to the complexity of the myocardial microcirculation, the theory of whether functional CMD is a precursor to structural pathologies has not yet been proven, requiring thorough exploration in the future.

In this study, there was a moderate correlation between rest T1 value and CVF, and an increase in CVF was consistent with the raised rest T1 value in the 15-week T2DM group. These results are consistent with prior studies which reported that rest T1 mapping can detect diffuse myocardial interstitial fibrosis (41, 42). Besides, another association was found between rest T1 value and MVD, which may be related to inflammation-induced microvascular rarefaction (43). The number of micro-vessels decreases with the development of inflammation and edema, as evidenced by an increase in T1 value (44). However, the rest T1 value results from a combination of factors, and lipid deposition, in turn, leads to a shorter T1 relaxion time (45), so an investigation is needed to determine whether T1 mapping can reflect myocardial microcirculation statue.

It’s discovered that the T1 values reported in this study are a bit higher than what is normally reported for a standard MOLLI sequence on a three T system, for the following potential explanations. It is commonly established that the heart-rate-dependent sampling scheme is helpful for avoiding underestimation of T1 times. For the purpose of our study, the MOLLI sequence’s acquisition interval was raised to 20 cardiac cycles in order to ensure full longitudinal magnetization recovery during the break between two acquisition blocks at higher heart rates. Zhao et al. employed the optimized MOLLI sequence among the AF patients and found that the mean myocardial T1 values in the population cohort were 1311.00 ms ± 44.5, a result consistent with our study’s (21). Additionally, by establishing a diabetic rabbit model, Zeng et al. measured the mean native T1 value in the myocardium of the interventricular septum at mid-SAX as 1,309 ms ± 91 and 1,324 ms ± 75 in the DM 6 months and 9 months groups, respectively, with a broader variety (46). In an animal study involving porcine as a model, the native T1 values of infarcted myocardium even reached 1,637 ms ± 123 and 1,471 ms ± 98 in the subacute vs. chronic phases, respectively (47). It is speculated that the elevation of T1 relaxation time may be associated with diffuse myocardial interstitial fibrosis and oedema, as verified by the pathological image presented in our study. The exact causes for T1 elevation in the animal study are yet to be elucidated.

### Limitations

The current study is subject to several limitations. First, although we optimized the T1 mapping sequence parameters by increasing the interval time from 3 to 20 cardiac cycles to complete the recovery of T1 longitudinal relaxation, image quality was inevitably affected, resulting in a slightly higher T1 value. Second, microvascular vasodilation mediated by ATP is non-endothelium-dependent. We cannot exclude the fact that endothelium-dependent abnormalities in coronary blood flow may influence the study results. Third, although we determined the correlation of the stress T1 response derived from CMR with pathological parameters, IMR and CFR obtained from PCI were not measured. Finally, this study was implemented on an animal model with a small sample size, and further validation with a larger T2DM cohort is needed in the future.

## Conclusion

In conclusion, our results indicate that stress T1 responses determined by CMR T1 mapping correlate well with histopathologic measures in T2DM rabbits and may be utilized as a sensitive tool for the evaluation of CMD-related myocardial damage in early T2DM.

## Data availability statement

The original contributions presented in this study are included in the article/Supplementary material, further inquiries can be directed to the corresponding authors.

## Ethics statement

This animal study was reviewed and approved by the Laboratory Animal Welfare and Ethics Committee of the Army Military Medical University.

## Author contributions

JW, YS, and PM contributed to the study concepts, study design, and coordination responsibility for the research activity planning. PM contributed to the data analysis and drafting of the manuscript. YH contributed to the critical advice on study design. JL, YH, LC, HL, and XZ contributed to the data analyses and interpretation. All authors contributed to the article and approved the submitted version.
