# Supplementary material for: Stress CMR T1-mapping technique for assessment of coronary microvascular dysfunction in a rabbit model of type II diabetes mellitus: Validation against histopathologic changes
Source: Front Cardiovasc Med. 2023 Jan 20;9:1066332. doi: 10.3389/fcvm.2022.1066332 (PMC9895118; doi:10.3389/fcvm.2022.1066332)
Supplement: Supplementary file 1 [file Table_1.DOCX]

## Supplementary Figure


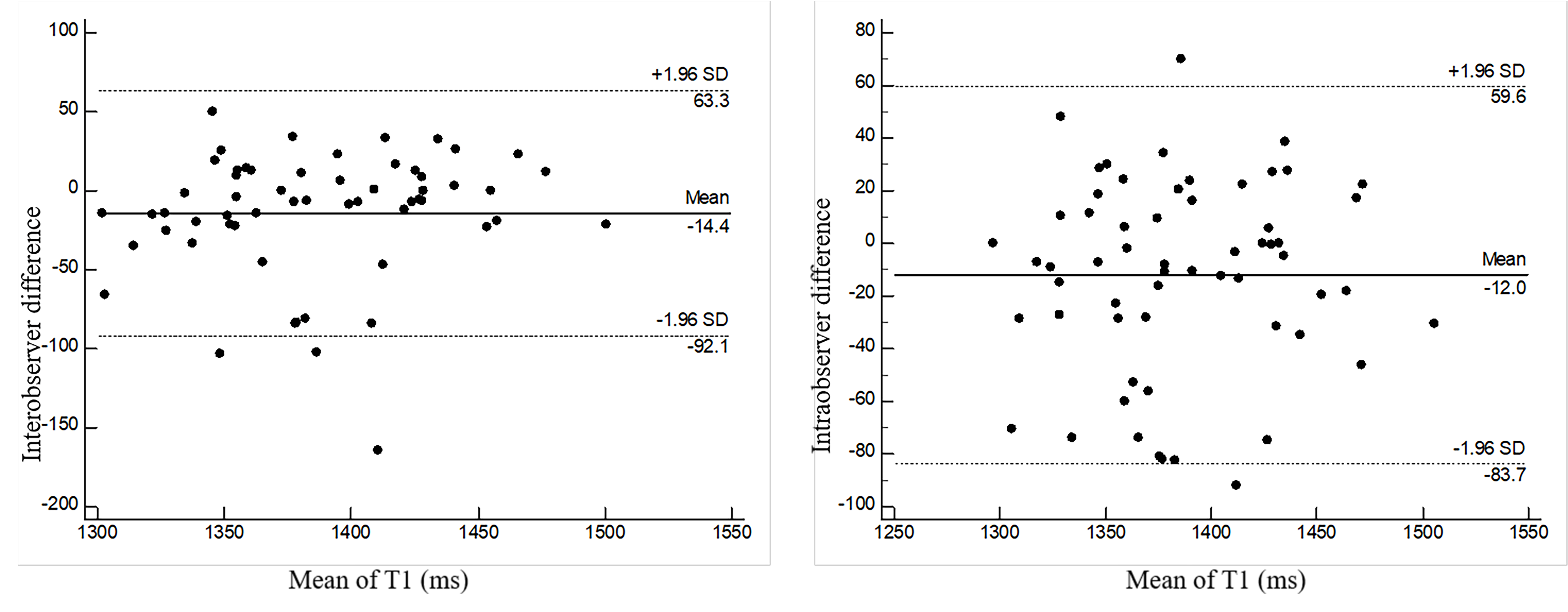


**Supplementary Figure 1.** Bland–Altman analysis of rest and stress T1 mapping


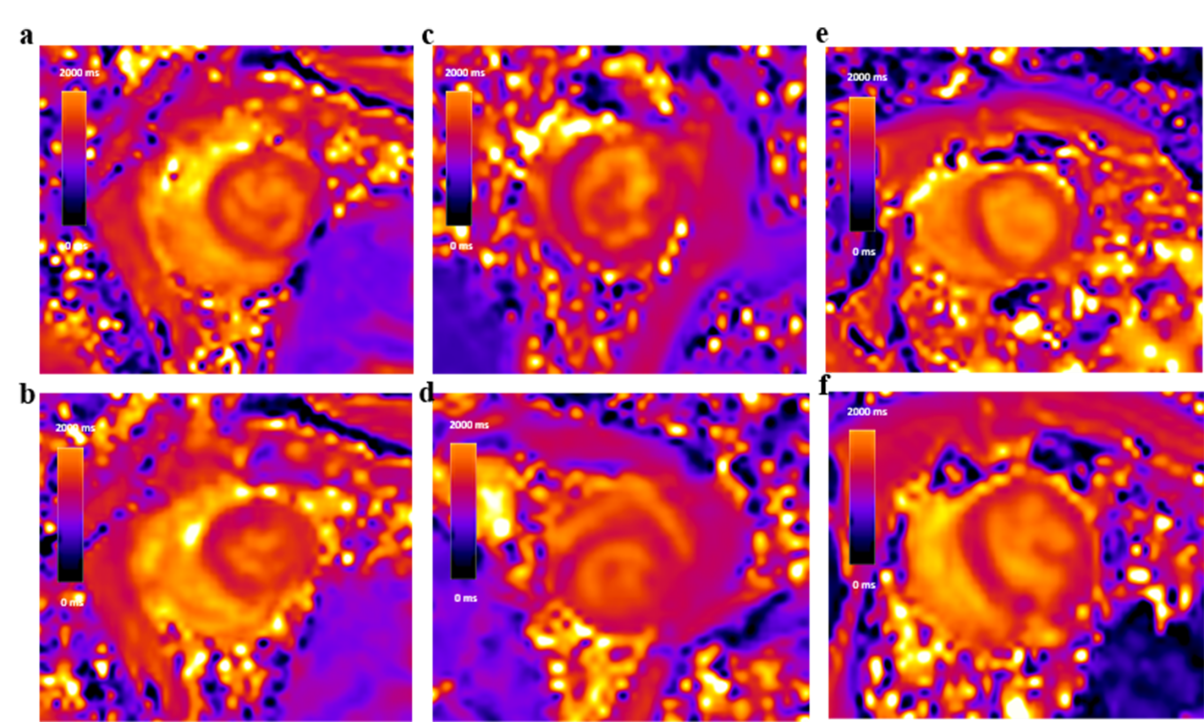


**Supplementary Figure 2.** Schematic representation of rest and stress T1 maps. A control rabbit with a resting T1 map, 1355ms **(a)** and corresponding stress T1 map, 1445ms **(b)**; A 5-week T2DM rabbit with a resting T1 map, 1321ms **(c)** and corresponding stress T1 map, 1379ms **(d)**; A 10-week T2DM rabbit with a resting T1 map, 1378ms **(e)** and corresponding stress T1 map, 1426ms **(f)**; Values in the graphs indicate the median T1 values of myocardial interventricular septum.
